# Evaluation of *Candida* colonization index, molecular identification, and antifungal susceptibility pattern of *Candida* species isolated from critically ill pediatric patients: A single-center study in Iran

**DOI:** 10.32598/CMM.2023.1372

**Published:** 2022-12

**Authors:** Amirhossein Davari, Mohammad Taghi Hedayati, Jalal Jafarzadeh, Bahram Nikmanesh, Mojtaba Nabili, Amir Ali Hamidieh, Mahdi Abastabar, Nasim Ahmadi, Abdullah M.S. Al-Hatmi, Maryam Moazeni

**Affiliations:** 1 Student Research Committee, Mazandaran University of Medical Sciences, Sari, Iran; 2 Invasive Fungi Research Center, Communicable Diseases Institute, Mazandaran University of Medical Sciences, Sari, Iran; 3 Department of Medical Mycology, School of Medicine, Mazandaran University of Medical Sciences, Sari, Iran; 4 Department of Medical Mycology and Parasitology, School of Medicine, Babol University of Medical Sciences, Babol, Iran; 5 Department of Medical Laboratory Sciences, School of Allied Medical Sciences, Tehran University of Medical Sciences, Tehran, Iran; 6 Department of Medical Laboratory Sciences, Faculty of Medicine, Sari Branch, Islamic Azad University, Sari, Iran; 7 Pediatric Stem Cell Transplant Department, Children’s Medical Center, Tehran University of Medical Sciences, Tehran, Iran; 8 Nosocomial Infection Medical Research Centre, Mazandaran University of Medical Sciences, Buali Sina Hospital, Sari, Iran; 9 Natural and Medical Sciences Research Center, University of Nizwa, Nizwa, Oman; 10 0Center of Expertise in Mycology of Radboud UMC/Canisius Wilhelmina Hospital, Nijmegen, The Netherlands

**Keywords:** Antifungals, *Candida* colonization index, Candidiasis, Pediatric

## Abstract

**Background and Purpose:**

Given the high mortality rate of invasive candidiasis in hospitalized pediatric patients, it is crucial to establish a predictive system to achieve early diagnosis and treatment of patients who are likely to benefit from early antifungal treatment. This study aimed to assess the *Candida* colonization index, species distribution, and antifungal susceptibility pattern of *Candida* strains isolated from pediatric patients with high *Candida* colonization index (CI).

**Materials and Methods:**

This study was carried out at the Children’s Medical Center in Tehran-Iran. In total, 661 samples were collected from 83 patients. The *Candida* CI was calculated according to the descriptions of previous studies. The isolates were identified using polymerase chain reaction-based techniques. The Clinical and Laboratory Standard Institute protocol M60 was used to conduct the antifungal susceptibility test.

**Results:**

A colonization index greater than 0.5 was confirmed in 29 cases (58% of positive samples) with two children developing candidemia. *Candida albicans* (n=53, 49.5%) was the most common *Candida* species in patients with CI > 0.5. Except for acute lymphoblastic leukemia, no risk factors were linked to a high index in colonized children (*P* > 0.05). Twelve isolates (7.01%) were multi-azole resistant with high MICs against both isavuconazole and ravuconazole and seven strains (4.09%) were echinocandins resistant.

**Conclusion:**

In pediatric intensive care units, patients are at risk of fungal infection, particularly candidemia. In this study, more than half of the children with positive yeast cultures had CI > 0.5, and 6.8% developed candidemia.

## Introduction

Candidemia is the second foremost cause of sepsis-related mortality in children [ 1 - 3 ]. Several publications have reported that the candidemia epidemiology, control, and outcome of candidemia differ noticeably and cannot be generalized to children [ 4 ]. Furthermore, given the high mortality rate of invasive candidiasis in intensive care unit (ICU) patients, it is crucial to establish an alarm system against this pathogenic yeast to achieve early diagnosis and treatment [ 5 ]. 

Understanding the link between the *Candida* colonization index (CI) and invasive candidiasis (IC) has been a priority for clinicians for decades; therefore, the colonization index has been proposed as the "missing link" and one of the most widely used scores is *Candida* CI [ 5 ]. Pittet et al. demonstrated that the *Candida* CI expresses the intensity of colonization and is the ratio of the number of non-blood distinct body sites screening positive for *Candida* species to the total number of distinct body sites tested [ 2 ].

In fact, multiple-site colonization with *Candida* spp. colonization is widely established as a key risk factor (5-30% of colonized patients) [ 6 , 7 ] for invasive fungal infection in seriously ill patients. Hence, a prognostic value, which could be colonization density, is needed for the diagnosis of systemic candidiasis [ 8 , 9 ]. *Candida* colonization has been reported in up to 60% of critically ill pediatric patients after 4-7 days in the ICU [ 10 - 12 ]. Early empirical management of acute candidiasis improves the chance of survival but might lead to antifungal overuse, which increases financial costs and also resistance in *Candida* isolates [ 13 ]. 

Adult patients and specific groups of diseases have been the focus of studies on this score. As a result, this study aimed to demonstrate the applicability of this score in pediatric patients at great risk for invasive candidiasis in pediatric/infant ICU (PICU and IICU) as well as in bone-marrow transplantation (BMT) units. The antifungal susceptibility profile of strains isolated from pediatric patients with high *Candida* colonization index was also determined.

## Materials and Methods

### Patients and sampling

**Chart 1:**
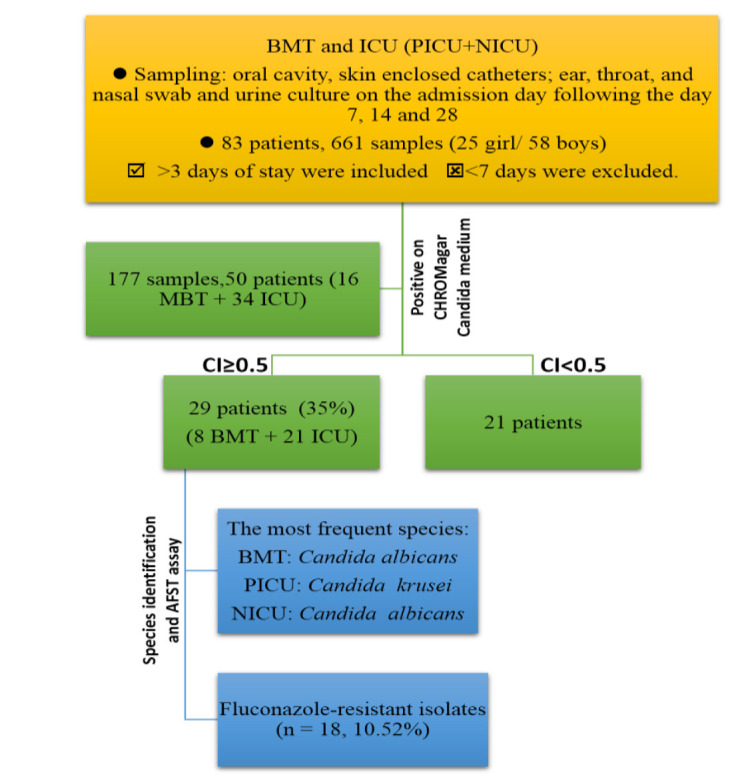
General flowchart of the present study. BMT: bone-marrow transplantation, ICU: intensive care unit, PICU: pediatric intensive care unit, NICU: neonatal intensive care unit

This research was performed at the Children's Medical Center in Tehran at the ICU and BMT unit from March 2021 to September 2021. In total, 661 samples were gathered from 83 patients. The age of patients ranged from newborn to 21 years old (). The patients who were supposed to stay for > 3 days were included in this study, from which, individuals with less than 7 days of hospitalization were excluded (See chart 1). This study was approved by the Ethics Committee of Mazandaran University of Medical Sciences (Code IR.MAZUMS.REC.1397.2771). Written informed consent was obtained from parents.

Samples from *Candida* colonization-prone body sites, such as the oral cavity, skin-enclosed catheters, ear, and throat, as well as nasal swab and urine culture were collected at the time of admittance and also every week during their stay. Swab samples were obtained by using swab sticks whereas urine samples were collected as mid-stream urine or via a urinary catheter. On the admission day and days 7, 14, and 28, the *Candida* colonization index was calculated for each patient. 

All samples were cultured on Sabouraud dextrose agar (HIMEDIA, India) with chloramphenicol as well as chromogenic medium (CHROMagar *Candida*, CHROMagar Microbiology, France). Each bottle contained a blood sample from a patient suspected of having candidemia which then was incubated at 35 ⁰C in the automated BACTEC™ 9120 blood culture system (Becton Dickinson, USA).

### Identification of the isolates

Primary as well as multiple-species identification was done by using chromogenic CHROMagar™ *Candida* medium (CHROMagar™, France). Genomic DNA was extracted [ 14 ] and a previously described polymerase chain reaction-restriction fragment length polymorphism (PCR-RFLP) method was applied to identify common *Candida* species [ 15 ]. Briefly, the internal transcribed spacer (ITS) 1-5.8S rDNA-ITS2 region was amplified using the primers ITS1 (5’- TCC GTA GGT GAA CCT GCG G-3’) and ITS4 (5’-TCC TCC GCT TAT TGA TAT GC-3’). 

The amplifications were conducted based on the following: initial denaturation at 95 ⁰C for 5 min followed by 35 cycles of denaturation at 95 ⁰C for 15 s, annealing at 56 ⁰C for 30 s, and elongation at 72 ⁰C for 30 s. After the final cycle, the samples were incubated for 5 min at 72 ⁰C [ 16 ]. The PCR products were first run on 1.2% agarose gels, visualized with UV after ethidium bromide staining, and then were digested with 5 U of the restriction enzyme MspI (Fermentas, Vilnius, and Lithuania) followed by gel electrophoresis on 2% agarose gel. *Candida albicans* and *Candida* parapsilosis species complexes were distinguished by amplification of the Hyphal wall protein and intein-containing vacuolar ATPase precursor genes, respectively, using previously described protocols [ 17 , 18 ]. 

### Candida colonization index evaluation

The *Candida* colonization index was analyzed as the proportion of the number of colonized body sites (other than blood culture) with *Candida* spp. upon the total number of cultured sites. Colonization was considered high when CI ≥ 0.5 [ 2 ]. 

### Antifungal Susceptibility Testing

Antifungal susceptibility testing (AFST) was performed based on the guidelines of the Clinical and Laboratory Standards (CLSI), M27-A3, and M60 [ 19 , 20 ]. Fluconazole (FLZ), itraconazole, voriconazole (VRZ), ravuconazole (RVZ), and isavuconazole (ISZ) were used as azole antifungals. Caspofungin (CAS) and anidulafungin (AFG) were applied as echinocandins and amphotericin B was the representative of polyene antifungals. All antifungal agents were from Sigma-Aldrich, Germany, and dissolved in dimethyl sulfoxide. Afterward, they were diluted in a standard RPMI 1640 medium (Sigma Chemical Co.) buffered to pH 7.0 with 0.165 3-(N-Morpholino) propane sulfonic acid (MOPS, Sigma Chemical Co.) with l-glutamine and without bicarbonate to yield their two folds initial concentrations. 

A suspension of fresh yeast colonies was prepared in a sterile saline solution with a transmittance range of 75-77%. A working suspension was made by 1:100 dilution which resulted in 2.5-5×10^3^ CFU/ml. Drug-free and yeast-free wells were included as positive and negative controls, respectively. The microdilution plates were incubated at 35 °C and examined visually after 24 h. *Candida krusei* (ATCC6258) and *C. parapsilosis* (ATCC 22019) were used as quality controls.

### Statistical analysis

The data were recorded in Microsoft Excel 2007 (Microsoft Corp, Redmond, WA, USA) and SPSS software (version 16; SPSS Inc., Chicago, IL, USA) was used for the analysis. Categorical variables were compared using the Fisher exact test to determine differences in their dispersals among the subgroups. Student’s t-test was applied to evaluate the geometric means yielded from antifungal susceptibility testing (AFST). The *P* values less than 0.05 were considered statistically significant.

## Results

A total of 206 yeast isolates were collected from different body sites of patients. The majority of the yeast isolates (n=104) were obtained from the urine. Other isolates were extracted from blood (n=52), vaginal swabs (n=45), ear discharge (n=2), tracheal aspirate (n=2), tissue (n=2), skin (n=1), nail (n=1), sputum (n=1), and cerebrospinal fluid (n=1). Among 206 yeast isolates, 200 isolates were identified as single species, and the most dominant species was *C. albicans*, with a number of 120 (59%). Out of 206 yeast cultures, six isolates were identified as a mixture of different *Candida* species by the PCR-RFLP method. Three mixtures combined *C. albicans* and *C. glabrata* (MY 1, MY 2, and MY 3). The fourth was the mixture of *C. glabrata* and *C. tropicalis* (MY 4), the fifth mixture combined *C. parapsilosis* and *C. tropicalis* (MY 5), and the sixth mixture was the combination of *C. glabrata*, *C. albicans*, and *C. tropicalis* (MY 6) .

### Candida colonization index evaluation

This study was performed on 83 patients (children/infants) from ICUs as well as BMT units. Overall, 661 swab and urine cultures were obtained from 83 patients. It should be noted that 177 out of 661 samples were collected from 50 individuals (26.8%) and were considered positive cultures with the least grown yeasts from one sample site. Moreover, 25 and 58 samples were isolated from female and male patients, respectively. 

The most reported sites with yeast growth were the oral cavity, ear, and nasal samples. In total, 50 (16 BMT+34 ICU) patients had yeast growth and the CI was found to be ≥ 0.5 in 29 (8 BMT+21 ICU) of them (58% and 35% of positive and total samples, respectively). It was found that colonization occurred most strongly two weeks after admission. Table 1 summarizes the demographic data of patients with CI > 0.5. Among pediatric patients with CI > 0.5, 12 patients (ICU: 7/21, 33.3%, BMT: 5/8, 62.5%, total: 12/29, 41.4%) suffered from acute lymphoblastic leukemia (ALL) as the predisposing factor, whereas this parameter was reported in three patients. Therefore, the chance of *Candida* colonization with CI > 0.5 in pediatric patients who suffered from ALL is significantly higher (Odd’s ratio with 95% CI was 4.4, *P*<0.05). 

**Table 1:** Demographic data of patients and distribution of the yeast species with colonization index ≥ 0.5.

Underlying Disease			Gender	Age (year and month)	Colonization index	Number of Samples	Positive Samples	Identified Candida Species
IICU and PICU units	1	COPD	M	2	0.7 week 0	9	8	*C. parapsilosis*, *C. albicans* *C. dubliniensis*, *C. krusei*
	2	Asthma	M	1	0.6-0.5 week 1-2	14	4	*C. krusei*, *C. guilliermondii*
	3	ALL	F	5	0.7-0.5 week 0- 1and 2	13	7	*C. krusei*, *C.albicans* *C. guilliermondii*
	4	ALL	F	7	0.5-0.6-0.4 week 0-1-2	14	7	*C. albicans*,*C. parapsilosis*
	5	VSD	F	3 m	0.5 week 0	4	2	*C. parapsilosis*
	6	LaryngomalaciaM	2 m	0.5 week 0 and 1	8	4	*C. africana*
	7	ALL	M	10	0.6-1 week 0-1-2	20	17	*C. krusei*, *C. albicans* *C. parapsilosis*, *C. lusitania*
	8	Distress Breathing	F	6 m	0.5-0.7 week 0-7	8	5	*C. albicans*, *C. krusei*
	9	ALL	F	3 m	0.4-0.5-0.6 week 0-1-2	14	8	*C. albicans*, *C. galbrata*
	10	Distress Breathing	F	10	0.5 week 0	4	2	*C. krusei*
	11	ALL	M	5	0.6-0.8 week 2-	15	7	*C. krusei*,*C.albicans* , *C. dubliensis*
	12	Seizure	M	8 m	0.5 week 1	8	2	*C. krusei*
	13	Pneumonia	F	4 m	0.5 week 1	8	2	*C. parapsilosis*, *C. dubliniensis*
	14	Meningitis	M	11	0.5 week 1	9	2	*C. glabrata*
	15	Asthma	F	4 m	0.5 week 4	24	2	*C .glabrata*
	16	ALL	F	9 m	0.5 week 0 and 1	11	4	*C. dubliniensis*
	17	Bronchitis	M	3 m	0.6-0.8 week 0-1-2-3	18	11	*C. krusei*, *C. parapsilosis*, *C. glabrata*
	18	ALL	M	14	0.6 week 1,2,3,4	18	10	*C. albican*, *C .dubliniensisn*, *C. glabrata*, *C. krusei*, *C. parapsilosis*
	19	Down Syndrome	M	9 m	0.5 week 1	4	2	*C. krusei*, *C. parapsilosis*
	20	Thalassemia	M	12 m	0.6 week 1	10	5	*C. parapsilosis*, *C. krusei* *C. dubliniensis*, *C .albicans*
	21	CVID	M	2	0.5 week 0 and 1	8	4	*C. parapsilosis*, *C.albicans*, *C. krusei*
BMT unit	22	ALL	M	5.5	0.5- week 2	8	2	*C. dubliniensis*
	23	ALL	M	11	0.5- week 2	8	2	*C. albicans*
	24	ALL	M	5	0.6- week 2	8	3	*C. albicans*, *C. parapsilosis*
	25	ALL	M	10	0.9- week 1 and 2	9	9	*C. albicans*
	26	Metabolic	M	1.5	0.5- week 2	4	2	*C. albicans*
	27	SCID	M	3.6	0.5- week 2	7	2	*C. parapsilosis*
	28	CGD	F	6	0.6- week 1	5	3	*C. albicans*
	29	ALL	F	5	0.5- week 2 and 3	13	5	*C. albicans*, *C. glabrata*

COPD: chronic obstructive pulmonary disease, ALL: acute lymphoblastic leukemia, VSD: ventricular septal defect, CVID: common variable immunodeficiency, SCID: severe combined immunodeficiency, CGD: chronic granulomatous disease, F: female, M: male

The CI > 0.5 was reported most in male patients, compared to female patients (n=18, 62% vs. n=11, 38%). However, being male or female did not significantly increase the possibility of Candida colonization occurrence with CI > 0.5 (Odd’s ratio 1.27, *P*>0.05 for males and 0.81, *P*>0.05 for females, respectively). In total, 11 patients (38%) were below one year old (considered infant); nevertheless, age was not observed as a significant effective parameter as the values of odd’s ratio values were 0.66 (*P*>0.05) and 1.5 (*P*>0.05) for pediatric patients over and under one year old, respectively. 

It is noteworthy that two (2/29, 6.8%) out of the total patients with CL>0.5 who suffered from ALL (see the demographic data of patient no. 11 in Table 1) and thalassemia (see the demographic data of patient no. 20 in Table 1) developed candidemia during weeks one and three, respectively. Regarding molecular species, identification, and AFST results, multi-azole-resistant *Candida albicans* and FLZ-resistant *C. parapsilosis* were isolated from blood culture bottles, respectively.

### Identification of the isolates

*Candida albicans* was the most common species overall, accounting for 53 strains (29.9%) out of 177 positive-grown samples. Notably, *C. krusei* was the most common species in PICU, accounting for 24 out of 61 strains (40%) and ranking second in IICU (18 out of 74 stains, 24%). Regarding the species distribution of BMT, *C. albicans*, *C. parapsilosis*, *C. dubliniensis*, *C. glabrata*, and *C. krusei* were detected in 59.5% (n=25), 26.3% (n=11), 7.2% (n=3), 4.7% (n=2), and 2.3%(n=1) of patients, respectively. 

Moreover, regarding the frequency of the species in IICU, *C. albicans*, *C. krusei* *C. parapsilosis*, *C. dubliniensis*, *C. glabrata*, *C. guilliermondii*, *C. lusitaniae*, *C. africana*, and *C. kefyr* were detected in 27% (n=20), 24%.(n=18), 16% (n=12), 10% (n=7), 7% (n=5), 7% (n=5), 5% (n=4), 3% (n=2), and 1% (n=1) of patients, respectively. In PICU, the *Candida* species, including *C. krusei*, *C. parapsilosis*, *C. dubliniensis*, *C. albicans*, and *C. glabrata* were found in 40% (n=24), 18% (n=11), 16% (n=10), 13% (n=8), and 13% (n=8) of patients, respectively. In patients with CI > 0.5, 107 strains were identified successfully. *Candida albicans* 49.5% (n=53) was the most frequent species followed by *C. glabrata* 18.7% (n=20) and *C. krusei* 14% (n=15) (Table 1).

### Antifungal Susceptibility Testing

Table 2 summarizes the minimum inhibitory concentration (MIC) range, MIC50, MIC90, and geometric mean (GM) MIC of eight antifungal drugs against identified different species. Six isolates were unable to be recovered from culture media; therefore, 171 isolates were subjected to AFST. Fluconazole-resistant isolates (n=18, 10.52%) were identified using the new breakpoints [ 20 ]. *Candida albicans* (n=12), *C. parapsilosis* (n=2), *C. glabrata* (n=1), *C. krusei* (n=2), and *C. guilliermondii* (n=1) were among the strains. Two *C. dubliniensis* strains had high MICs against FLZ (0.5-1µg/ml) based on epidemiological cutoff values (ECVs). Among FLZ-resistant strains, 11 strains of *C. albicans* and one *C. glabrata* isolate were multi-azole resistant (7.01%) and had high MICs against both ISZ and RVZ. 

**Table 2:** Geometric mean, MIC50, and MIC90 values were obtained by testing the susceptibility of isolated *Candida species*. (MIC50 and MIC90 of the isolated species of less than 5 were not calculated)

Antifungal agent	Candida species	MIC range (µg/ml)		MIC50 (µg/ml)		MIC90 (µg/ml)		GM (µg/ml)	
		ICU	BMT	ICU	BMT	ICU	BMT	ICU	BMT
Amphotericin	*C. albicans*	0.125-4	0.5-2	0.5	1	2	2	0.6771	1.1626
	*C. dubliniensis*	0.25-1	0.5	0.5		1		0.5612	0.5
	*C. parapsilosis*	0.5-2	0.5-2	0.5	1	2	2	0.6597	1.2599
	*C. glabrata*	0.5	0.5					0.5	0.5
	*C. krusei*	0.5-2	2	0.5		0.5		0.7348	2
	*C. guilliermondii*	0.125-0.5		0.5		2		0.3789	
	*C. africana*	0.25						1	
	*C. lusitaniae*	1						0.5	
Fluconazole	*C. albicans*	0.125-16	0.031-16	1	0.25	4	16	0.6898	1.2721
	*C. dubliniensis*	0.125-1	0.125-0.5	0.5		1		0.4851	0.25
	*C. parapsilosis*	1-2	0.125-16	1	0.25	2	16	0.7578	1
	*C. glabrata*	1	4-16					1	5.656
	*C. krusei*	0.5-16	1	1		1		0.8203	1
	*C. guilliermondii*	1-8		1		8		0.3077	
	*C. africana*	0.125						0.125	
	*C. lusitaniae*	0.5						0.5	
Voriconazole	*C. albicans*	0.031-2	0.016-16	0.062	1	0.031	16	0.0777	0.6977
	*C. dubliniensis*	0.031-0.125	0.031-0.63	1		0.25		0.0609	0.0392
	*C. parapsilosis*	0.016	0.016-2	0.031	0.031	0.125	2	0.0415	0.0581
	*C. glabrata*	0.0125	16					0.0125	16
	*C. krusei*	0.016-0.25	0.125	0.016				0.0727	0.125
	*C. guilliermondii*	0.031-1		0.062		0.016		0.0474	
	*C. africana*	0.016				0.25		0.016	
	*C. lusitaniae*	0.031						0.031	
Itraconazole	*C. albicans*	0.0625-16	0.031-16	0.25	0.25	0.5	16	0.2614	0.7387
	*C. dubliniensis*	0.125-0.5	0.25	0.25		0.5		0.2024	0.25
	*C. parapsilosis*	0.125	0.063-16	0.125	0.125	0.125	16	0.1894	0.4397
	*C. glabrata*	0.25	16					0.25	16
	*C. krusei*	0.125-16	1	0.25		0.25		0.2973	1
	*C. guilliermondii*	0.125-0.25		0.25		0.5		0.203	
	*C. africana*	0.25						0.25	
	*C. lusitaniae*	0.5						0.5	
Ravuconazole	*C. albicans*	0.016-0.5	0.016-16	0.0625	0.125	0.25	16	0.0515	0.8131
	*C. dubliniensis*	0.016-0.0125	0.063-0.5	0.0625		0.25		0.0574	0.1256
	*C. parapsilosis*	0.0625-1	0.031-4	0.0625	0.125	1	4	0.0950	0.1987
	*C. glabrata*	0.031	0.063-16					0.031	1.003
	*C. krusei*	0.016-0.25	0.5	0.031		0.031		0.0514	0.5
	*C. guilliermondii*	0.0625		0.0625		0.016		0.0625	
	*C. africana*	0.25						0.25	
	*C. lusitaniae*	0.0625						0.0625	
Isavuconazole	*C. albicans*	0.016-1	0.031-16	0.031	1	0.0625	16	0.0364	0.7616
	*C. dubliniensis*	0.016-0.0625	0.031		0.031	0.0625		0.0331	0.031
	*C. parapsilosis*	0.031-0.0625	0.031-2	0.031	0.031	0.0499	1.2	0.0356	0.0783
	*C. glabrata*	0.016	0.25-16					0.016	1.414
	*C. krusei*	0.016-0.125	0.5	0.016		0.016		0.0328	0.5
	*C. guilliermondii*	0.016-0.031		0.031		0.0625		0.0291	
	*C. africana*	0.031						0.031	
	*C. lusitaniae*	0.0625						0.625	
Caspofungin	*C. albicans*	0.016-16	0.016-0.5	0.031	0.016	0.5	0.016	0.0537	0.0196
	*C. dubliniensis*	0.016-0.625	0.016-0.03	0.031		0.0625		0.0354	0.0199
	*C. parapsilosis*	0.016	0.016-1	0.016	0.016	0.016	1	0.016	0.1005
	*C. glabrata*	0.016	0.016					0.016	0.016
	*C. krusei*	0.016-16	16	0.016		0.016	0.0582		16
	*C. guilliermondii*	0.031-0.125		0.031		0.062		0.0359	
	*C. africana*	0.016						0.016	
	*C. lusitaniae*	0.31						0.31	
Anidulafungin	*C. albicans*	0.016-16	0.008-2	0.031	0.25	1	0.016	0.0629	0.0326
	*C. dubliniensis*	0.031	0.125-0.25	0.031		0.031	0.016	0.031	0.1574
	*C. parapsilosis*	0.031-0.125	0.016-2	0.0625	0.016	0.1		0.0475	0.1987
	*C. glabrata*	0.125	0.016					0.125	0.016
	*C. krusei*	0.016-4	8	0.125		0.125		0.0584	8
	*C. guilliermondii*	0.031-0.016		0.031		0.062		0.0239	
	*C. africana*	0.016						0.016	
	*C. lusitaniae*	0.016						0.016	

MIC: minimum inhibitory concentration. MIC50: minimal concentration that inhibits 50% of isolates, MIC90: minimal concentration that inhibits 90 % of isolates, GM: geometric mean,

ICU: intensive care unit, BMT: bone-marrow transplantation unit

In the case of echinocandins, 10 isolates (5.85%) were resistant to AFG, including *C. albicans* (n=3), *C. krusei* (n=3), and *C. parapsilosis* (n=4). Moreover, 9 isolates (5.26%) including *C. albicans* (n=1), *C. parapsilosis* (n=3), and *C. krusei* (n=4) were resistant to CAS. One, two, and four strains of *C. albicans*, *C. krusei*, and *C. parapsilosis*, respectively, were resistant to all echinocandins (total: 4.09%) (see Table 2). 

According to the new ECV (CLSI M59 2^nd^ Ed, 2018), five strains of *C. parapsilosis*, four strains of *C. krusei*, and nine strains of *C. albicans* had high MICs against AmB (10.52%). Table 2 provides additional information about the AFST performed on all isolated species. The GM of *C. albicans* isolates, which was reported to be the most prevalent isolated species, was found to be significantly different between the patients in the BMT unit and ICU (*P*=0.004). *Candida albicans* isolated from patients in ICU had higher GM against all tested antifungals, compared to *C. albicans* isolated from BMT units. According to MIC50 values of the species isolated from ICUs, CAS with a MIC value of 0.016 µg/ml was reported as the most effective agent.

## Discussion

The overall rate of fungal colonization (CI>0.5) was 35%. Therefore, it can be said that roughly, the majority of patients are at risk of improving their invasive candidiasis. As a result, one ALL patient (1/29, 3.4%) developed candidemia during week three. In line with the findings of this study, those of the research performed by Altintop et al. (2018) indicated a 33.3% *Candida* colonization rate in patients in PICU [ 5 ]. In their study, *C. albicans* was the predominant species followed by *C. parapsilosis* and *C. glabrata* *Candida albicans* (n=53, 29.77%), *C. krusei* (n=43, 24.16%), *C. parapsilosis* (n=34, 19.10%), and *C. glabrata* (n=15, 8.42%).

Furthermore, Hamzavi estimated the *Candida* colonization incidence rate at 59.9% (82/136) in patients with hematological malignancy and found that *C. albicans* (72.0%) was the most common *Candida* species in colonized patients. They also reported that *Candida* colonization did not have a relationship with age, gender, oncologic diseases, and degree of neutropenia [ 21 ]. The species distribution differed from the findings of a previous study conducted at the Children’s Medical Center in Tehran [ 15 ]. 

According to Charsizadeh et al., *Candida albicans* and *C. parapsilosis* were the most commonly isolated species from ICU patients with invasive candidiasis. Although *C. albicans* was the most common species overall in the current study, *C. krusei* was the most common species in patients in PICU. The high rate of *C. krusei* isolation from hospitalized pediatrics in PICU is cause for concern. It was also reported as the third most isolated species in patients with CI > 0.5. 

The patients in the BMT unit must stay in that unit for several days or even months; therefore, the number of involved patients is lower than that of the ICU unit. The most important reasons for the low rate of positive samples were prophylactic antifungal use and daily bathing. 

According to Solomkin et al., colonization at more than two sites has been previously proposed as a key to the earlier initiation of antifungal therapy in high-risk surgical populations. Previous studies also suggested that early systemic antifungal therapy may be beneficial in patients who have been colonized at more than two sites but do not have candidemia [ 22 , 23 ]. Based on AFST results, more than 10% of the isolates were FLZ-resistant. The selection of resistant species during prophylaxis and antifungal therapy, as well as the increased number of infections caused by drug-resistant fungal species and lack of satisfactory outcomes, highlight the need for more effective prevention and treatment strategies based on new antifungal drugs and fungal species-specific diagnoses. 

The main limitation of the current study was the relatively small number of enrolled pediatric patients and time constraints in terms of following up with the patients. More studies are needed to understand the impact of risk factors on CI values and the complicated journey of Candida spp. from colonization to infection in pediatric patients.

## Conclusion

In pediatric intensive care units, patients are at risk of fungal infection, particularly candidemia. In this study, more than half of the children with positive yeast cultures had CI > 0.5, and 6.8% developed candidemia.

## Acknowledgments

This research was supported by Mazandaran University of Medical Sciences, Sari, Iran [Grant No. 2771].

## Authors’ contribution

M. M. conceived of the study and wrote the manuscript. A. D., N. A., and J. J. collected samples and performed the experiments. A. H., M. H., M. N., and B. N. advised the whole process of the study. A. A. and M. A. edited the manuscript. All authors read and approved the final manuscript. 

## Conflicts of interest

There is no conflict of interest.

## Financial disclosure

The patients were aware of the purpose of the study and informed consent was obtained from each participant.
